# Correction: Reductive annulations of arylidene malonates with unsaturated electrophiles using photoredox/Lewis acid cooperative catalysis

**DOI:** 10.1039/d1sc90070f

**Published:** 2021-04-09

**Authors:** Rick C. Betori, Benjamin R. McDonald, Karl A. Scheidt

**Affiliations:** Department of Chemistry, Center for Molecular Innovation and Drug Discovery, Northwestern University 2145 Sheridan Road Evanston Illinois 60208 USA Scheidt@northwestern.edu

## Abstract

Correction for ‘Reductive annulations of arylidene malonates with unsaturated electrophiles using photoredox/Lewis acid cooperative catalysis’ by Rick C. Betori *et al.*, *Chem. Sci.*, 2019, **10**, 3353–3359, DOI: 10.1039/C9SC00302A.

In the original manuscript, the authors utilised DPAIPN as a photocatalyst for the development of a reductive annulation of arylidene malonates to form chromanes. The structural assignment of the prepared DPAIPN photocatalyst was made in comparison to the proton and carbon NMR data reported by Zhang *et al.*^1^ (ref. 16 in the original manuscript).

An article correction notice was recently published for this *ACS Catalysis* article by Zhang *et al.*,^1^ which outlines the discovery and use of carbzolyl dicyanobenzene donor–acceptor fluorophores that serve as metal-free photoredox catalysts in organic synthesis. In the *ACS Catalysis* correction, Zhang *et al.* noted that one of the catalysts described in their manuscript, “1,3-dicyano-2,4,5,6-tetrakis(*N*,*N*-diphenylamino)-benzene” (herein referred to as “4DPAIPN”) was not consistent with its characterization data and should be replaced with the catalyst name and structure for “2,4,6-tris(diphenylamino)-5-fluoroisophthalonitrile” (herein referred to as “3DPAFIPN”).

In light of this correction, Scheidt *et al.* conducted a follow-up structural characterisation (^19^F NMR and high-resolution mass spectrometry) of the “DPAIPN” catalyst batches used. Their data has led to the conclusion that the catalyst was improperly labelled as “DPAIPN” and instead should be labelled as “3DPAFIPN”. The obtained characterization data also matches that which was reported for “3DPAFIPN” by Zeitler *et al.*,^2^ the first instance of a structural correction being made for “4DPAIPN”.

This structural reassignment results in no changes to the electrochemical properties of the photocatalysts utilised in the original manuscript and thus does not alter the putative reaction mechanism reported in the manuscript.

Any instances of “DPAIPN” (referred to as “**7**” within the manuscript) should therefore be read as the correct catalyst name “3DPAFIPN” in the original manuscript. The Electronic Supplementary Information has been updated to reflect these changes.

The conclusions of the manuscript remain unaffected by the incorrectly labelled photocatalyst.

The Royal Society of Chemistry apologises for these errors and any consequent inconvenience to authors and readers.

## Supplementary Material
